# Clinical application values of a novel synthetic training simulator for bulbar urethral anastomosis

**DOI:** 10.1002/bco2.426

**Published:** 2024-08-30

**Authors:** Jing‐Dong Xue, Ping Zhang, Yue‐Min Xu, Ying‐Long Sa, Hui‐Quan Shu, Lin Wang, Hong Xie, Chao Li, Wei Zhang, Chao Feng, Deng‐Long Wu

**Affiliations:** ^1^ Department of Urology Tongji Hospital, School of Medicine, Tongji University Shanghai China; ^2^ Department of Reproductive Medicine The International Peace Maternity and Child Health Hospital, School of Medicine, Shanghai Jiao Tong University Shanghai China; ^3^ Shanghai Key Laboratory of Embryo Original Disease Shanghai China; ^4^ Department of Urology Shanghai Jiao Tong University Affiliated Sixth People's Hospital Shanghai China; ^5^ Department of Urology, Renji Hospital Shanghai Jiao Tong University School of Medicine Shanghai China; ^6^ Department of Urology, Tangdu Hospital Air Force Military Medical University Xi'an Shaanxi China

**Keywords:** bulbar urethra, learning curve, simulator, surgical training, urethral anastomosis, urethroplasty

## Abstract

**Purpose:**

This study aimed to report a newly developed, high‐fidelity synthetic simulator to simulate excision and primary anastomotic (EPA) bulbar urethroplasty and its clinical use for new practitioners in shortening the learning curve.

**Material and Methods:**

The bulbar urethral anastomosis simulator consists of several standardized components created according to the actual size of the male patient. Interns, novice residents, and fellows inexperienced with urethral reconstruction (*n* = 10, 5, 5) from different medical centres were invited to participate in the training programme. Two reconstructive urology experts monitored each practice. Following the training, three kinds of validity testing were used to assess the simulator: face, content, and construct. In the intern group, the task performance in the first five training sessions and the last five training ones were compared using a self‐control approach. In the resident and fellow group, the real surgical data, including estimated blood loss, operative duration, and 6‐month post‐operative success rate of trainees after training, are plotted, which are compared with that of reconstructive urology experts (*n* = 5) included retrospectively to study the effectiveness of the simulator in shortening the learning curve.

**Results:**

The overall mean satisfaction rate for the simulators was inspiring and evaluated by experts. In the intern group, significant improvement can be achieved through 10 training sessions (*p* < 0.05). In clinical practice, the intraoperative indicators and surgical success rate of both the training groups showed the tendency to close or even better than those in the expert group. In terms of the learning curve, training groups performed better compared with experts in the early stages of their careers.

**Conclusions:**

In conclusion, this synthetic training simulator for bulbar urethral anastomosis is novel, effective, and convenient for beginners of different groups. The training course can bridge the gap between preclinical use and actual surgery via this simulator.

## 1 INTRODUCTION

Due to the surgical difficulty and relatively high failure rate compared with other urological laparoscopic or open operations, urethroplasty is always one of the most challenging procedures for the reconstructive surgeon.^1^ Cultivating a reconstructive urologist requires a long cycle, and surgical proficiency in urethral reconstruction can be achieved after a learning curve spans nearly 100 cases.^2^ Errors of judgement, omission, wrong order, and technique can lead to surgical failure, decreased patient satisfaction, reoperation, permanent injury, and subsequent litigation. The lack of systematic training is the main factor that leads to new practitioners making mistakes and prolonging the learning curve in developing countries and those in developed countries.^3^, ^4^ Owing to this situation, simulation training was advocated to help the doctor master the critical steps of a procedure within a short period.^5^ Meanwhile, simulated task training can complement the historical surgical teaching simulator by allowing the trainees to master the technique without risking patient harm.

Over the last 100 years, the traditional training simulator has changed dramatically.^6^ Generally, an ideal surgical training simulator should contain several criteria, including mimicking the entire surgical procedure, providing accurate sensory feedback, and offering multiple copies of the simulator for rapid cycling through simulations to ensure that all trainees can practice the technique. Most importantly, the simulator should be conducive to improving trainee understanding of the procedures and increasing their confidence in operation.^7^

In recent decades, many procedure‐specific simulators have been reported for laparoscopic and robot‐assisted urological surgery. However, very few simulators have been produced and validated for open urological surgery, especially in urethroplasty. We developed a newly developed, high‐fidelity synthetic model to simulate EPA bulbar urethroplasty and reported its use for new practitioners in shortening the learning curve.

## 2 MATERIALS AND METHODS

### 2.1 Urethral anastomosis simulator design and creation

**FIGURE 1:**
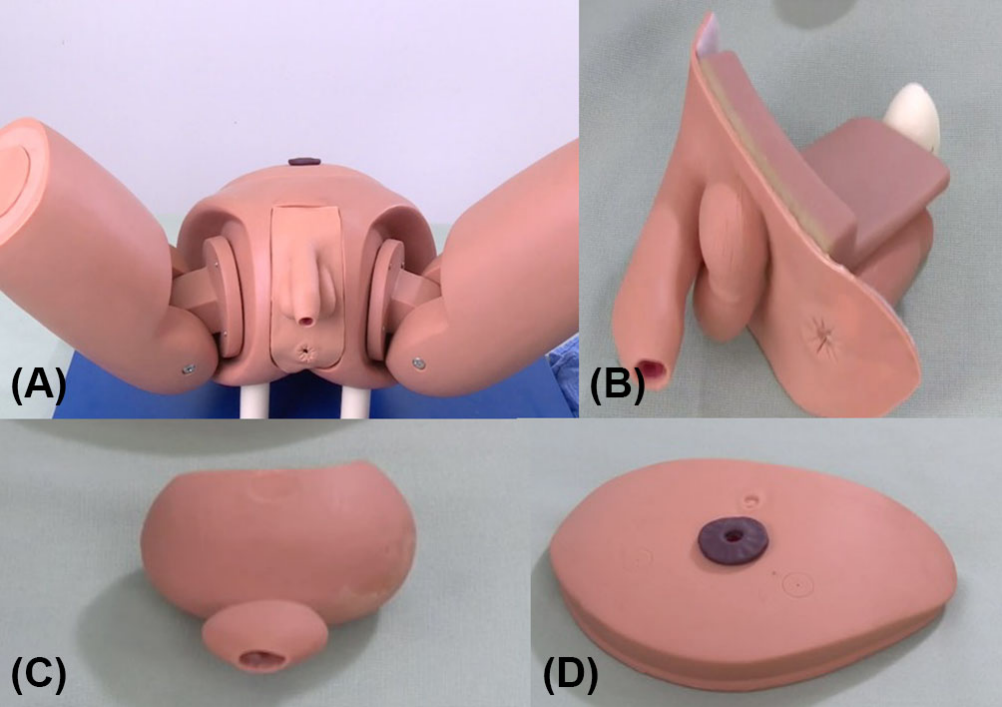
The holistic view of the simulator. The model consists of several standardized components including bony part of the pelvis extends to the low abdominal and upper thigh, urethra part, bladder part, and abdominal wall part.

The simulator consists of several standardized components created according to the actual size of the male patient (Figure 1). The bony part of the pelvis extends to the low abdominal and upper thigh. The low abdominal part is hollow for placing the bladder and abdominal wall parts. The upper thigh is movable so the trainee can adjust a suitable position for the procedure. In the middle of the perineal, another hollow structure is created for the urethra part. The bladder part is also open, which connects with a hollow prostate. The abdominal wall part can cover the low abdominal defect. In the centre of this part, a hole of cystostomy is created so that the sound can be inserted via this hole into the empty bladder and pass through the prostate and membranous urethral until the stricture site of the bulbar urethra. The urethral part is the key to the whole simulator. We used three different kinds of silicone to simulate the perineal skin, fat tissue, and muscle tissue around the urethra, respectively. The latex urethra was embedded into the urethral part according to the physiological curve of the male urethra. The site of simulated urethral stricture was standardized as length of 1 cm. The urethral inner cavity was sealed with a filling material, which was 6 cm away from internal end of urethral part. Besides these, we also simulated the rectum in the urethral part, which is located 0.5 cm beneath the urethra. The hollow rectum contained red colour fluid to indicate the possibility of rectal injury.

### 2.2 Participant recruitment

Interns, novice residents, and fellows inexperienced with urethral reconstruction (*n* = 10, 5, 5) from different medical centres were invited to participate. Participation was voluntary, and there were no participant exclusion criteria. A clear voluntary involvement announcement was made to the participants, and the trainees consented before participating in the study. Two experts (Xu YM and Sa YL) were invited to serve as judges and evaluate the simulator.

### 2.3 Validity testing in the training room

According to the traditional testing for simulators, we used three kinds of validity testing for assessment. Face validity relates to the degree of realism of the simulator concerning the actual anatomy and setup. Content validity involves the measurement of the appropriateness of the simulator as an effective training modality. Construct validity tests can reflect whether progress can be made through repeated training of the simulator.^8^

Based on the previous simulator's assessment, we redesigned a structured questionnaire for the face and content validity of the bulbar urethral anastomosis. At the end of the course, all experts (Xu YM and Sa YL) completed this questionnaire to assess the simulator. The evaluation of realism on (1) anatomy and colour, (2) sensation of texture and feeling of resection in each layer, (3) conductibility of urethral anastomosis, and (4) safety and efficacy of were the endpoints used for the assessment of the bulbar urethral anastomosis model. The following questions were used for content validity: “Was the model useful in teaching bulbar urethral anastomosis?” “Did you think the skills learned on the simulator are transferrable to the operating room?” “Did you feel more confident performing bulbar urethral anastomosis after practicing on the simulator?”

**FIGURE 2:**
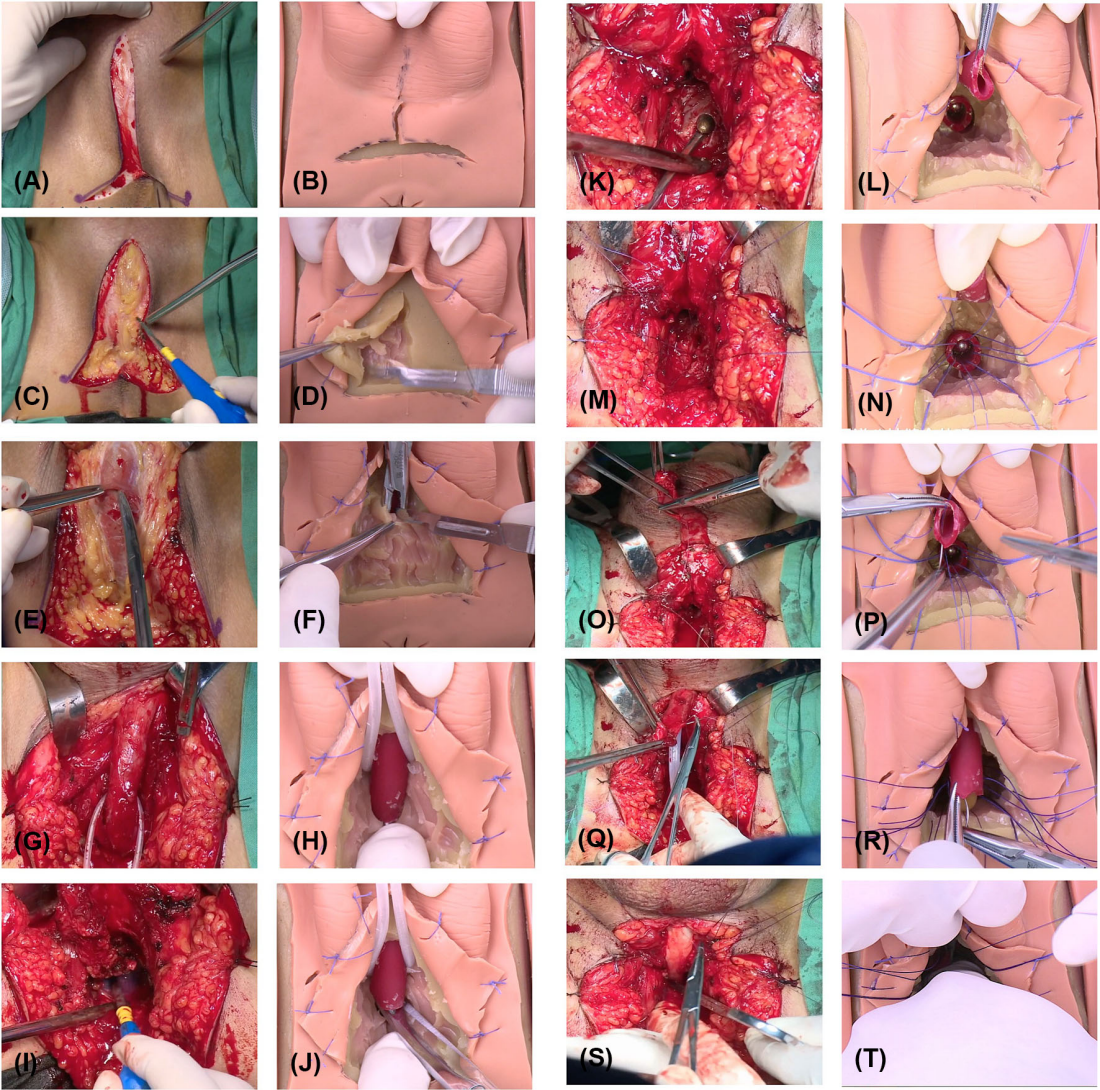
Surgical steps of simulated surgery versus real surgery. The procedure includes five key steps, the position of the inverted Y shape incision, exposition of the surgical incision, separation of the bulbar urethra, detection of urethral stricture, excision of urethral stricture, and urethral anastomosis.

For the construct validation, we compared the task performance of participants in the first five sessions and the last five sessions using a self‐control approach. Before practice, each participant viewed a 30‐min instructional video (Appendix S1, 4‐min video produced for the layout reduction version), and guidance for only first‐round was provided during course completion. Then, two experts (Xu YM and Sa YL) monitored each practice. Each participant had three urethral components for training. The length of stricture in the urethral part was 1 cm. The procedure was divided into five key steps, including the position of the inverted Y shape incision, exposition of the surgical incision, separation of the bulbar urethra, detection of urethral stricture, excision of urethral stricture, and urethral anastomosis (Figure 2). Especially the urethral end had to be anastomosed using six distributed sutures (Monocryl 3‐0). Each step was graded by both experts after discussion, from poor to excellent, quantified from one to 10 points with quantitative scores. The anastomosed urethral part was removed from the simulator and inspected. In the patency test, patency scales were defined as the maximal size of a catheter that could pass through the anastomosed urethra, in which the Fr10 catheter is equal to one score and the Fr18 catheter is equivalent to a five score. In the leakage test, the leakage was determined by injecting 20 mL of water from the external orifice of the urethra at a constant rate over 5 s. It was defined as the amount of water that exited at the anastomosis. The minimum and maximum values were 0 and 20 mL, respectively.

### 2.4 Assessment in the operation room

Following the first round of the training course, novice residents and fellows limited experience in urethral reconstruction were selected for direct clinical practice with patients. When each participant performed bulbar EPA, the estimated blood loss and operative duration were recorded. The 6‐month surgical success rate was followed as evaluation criterion. Additionally, the Global Rating Scale of operative performance (GRS) pointed by two experts (Xu YM and Sa YL) was also used to assess each operation.

### 2.5 Retrospective collection of expert data

Excluding the two judges mentioned, another five expert data from different medical centres were collected for the original learning curves. The collected data mainly includes the data of bulbar urethral EPA surgery in the early stages of their career, including surgical time, intraoperative blood loss, and 6‐month prognosis (surgical success rate). The included experts should have no less than 500 cases of urethral reconstruction surgery experience so far. Then these data are used to create learning curves as benchmark data for subsequent comparisons. Accordingly, we defined the learning curve as the time to reach a specified success rate (>90%).

### 2.6 Statistical analysis

Descriptive statistics were performed on demographic variables and presented by Origin, version 2021 (OriginLab Corporation, Northampton, MA, USA). Continuous variables were compared across the three groups using analysis of variance (ANOVA). Polytomous variables were compared across the three groups using nonparametric test. Post hoc testing was performed to determine which groups were statistically different. Differences in group means were analysed using one‐way analysis of variance tests. All p‐values were two‐sided and set to <0.05 for significance. All statistical analyses were performed using SPSS, version 25 (International Business Machines Corporation, Armonk, NY, USA).

## 3 RESULTS

The total cost of one simulator was $300. And each urethral training part costs $20.

Twenty individuals participated in the study and completed all research and evaluation components from January 2018 to December 2023. All participants were classified into three groups: intern, novice resident, fellows inexperienced with urethral reconstruction (*n* = 10, 5, 5). All these participants had no urethroplasty experience as the operation surgeon beyond training. Demographic and career surgical experienced are presented in Table S1.

For the outcome of face validity and content validity, the details are shown in Table S2. The overall extremely high satisfaction for the simulators was given by the two experts (Xu YM and Sa YL). In the content validation, all experts agreed that this simulator was a valuable and practical simulator for urethral anastomosis training. They all felt that this simulator could fix the gap between the training room and operation, and further expressed that this model could be used as a routine training model.

Table 1 demonstrates the scores of each step in the intern group for the construct validation. The result seems to indicate that significant improvement can be achieved through 10 training sessions. The performance of the last five training sessions had significantly improved compared with the first five ones, both in terms of fluency and outcome evaluations (*p* < 0.05). In the patency test, the anastomotic site could pass through the bigger catheter in the last five training sessions (*p* < 0.05). In the leakage test, less water exited from the anastomosed site in last five training sessions (*p* < 0.05).

**TABLE 1:** Results of construct validity of the urethral anastomosis simulator in the intern group (*n* = 10).

	First five training sessions	Last five training session	*p* value
Position of the inverted Y shape incision	7.92 ± 0.76	9.44 ± 0.51	0.000
Exposition of the surgical incision	7.24 ± 0.78	8.93 ± 0.64	0.000
Separation of the bulbar urethra	6.40 ± 1.03	8.88 ± 0.72	0.000
Detection of urethral stricture	6.20 ± 1.18	9.16 ± 0.68	0.000
Excision of urethral stricture	5.56 ± 1.12	8.63 ± 1.23	0.000
Urethral anastomosis	4.84 ± 0.85	8.49 ± 1.08	0.000
Patency	6.28 ± 0.79	8.39 ± 0.96	0.000
Leakage test (mL)	7.48 ± 2.48	2.80 ± 1.28	0.000

*Note*: All statistics are reported as mean ± (SD).

A total of 250 consecutive cases bulbar urethral EPA surgery underwent from five experts (50 cases per one expert) in the early stages of their careers were analysed, with an overall functional success rate of 88.0%. Demographic variables of the patients are presented in Table 2. The case number to reach proficiency (>90% success, <60‐min operation time, <100‐mL estimated bleeding loss), was approximately 18–20 cases for this type of reconstruction for bulbar urethroplasties (Figure S1). Among these 50 surgical data, the first 25 cases and the last 25 cases were used for comparative analysis (Table 3). The results showed that the learning curve of this type of surgery can be explained by the data of the first 25 cases, regardless of surgical time, bleeding volume, and surgical success rate.

**TABLE 2:** Comparison of surgical manifestations of the first 25 cases in three groups.

	Expert (*n* = 5)	Fellow (*n* = 5)	Resident (*n* = 5)	*p* value
**Preoperation patients' characters**				
Case no.	125	125	125	
Patients age	43.13 ± 12.40	43.84 ± 14.18	45.66 ± 14.71	0.329
**Aetiology**				
Straddle injury	89	75	72	0.184
Iatrogenic injury	28	39	44	
Idiopathic or not mentioned	8	11	9	
**Assessment during operation**				
Length of stricture (cm)	1.89 ± 0.47	1.95 ± 0.44	1.93 ± 0.45	0.607
Estimated blood loss (mL)	161.92 ± 62.81	141.84 ± 48.99	168.64 ± 59.61	0.001
Operation duration (min)	100.64 ± 26.49	90.20 ± 22.88	99.32 ± 28.51	0.006
Total success rate 6‐month after surgery	88.0%(110/125)	89.6%(112/125)	91.2%(114/125)	0.973

**TABLE 3:** Results of bulbar EPA of expert group (*n* = 5) in operation room.

	25 before	25 after	50 total	*p* value*
**Preoperation patients' characters**				
Total case no.	125	125	250	
Patients age	43.13 ± 12.40	42.29 ± 12.55	42.71 ± 12.45	0.599
**Aetiology**				
Straddle injury	89	73	172	0.369
Iatrogenic injury	28	35	63	
Idiopathic or not mentioned	8	7	15	
**Assessment during operation**				
Length of stricture (cm)	1.89 ± 0.47	1.93 ± 0.44	1.91 ± 0.46	0.533
Estimated blood loss (mL)	161.92 ± 62.81	87.20 ± 28.67	124.56 ± 61.44	0.000
Operation duration (min)	100.64 ± 26.49	70.76 ± 8.98	85.70 ± 24.77	0.000
Total success rate 6‐month after surgery	88.0%(110/125)	95.2%(119/125)	91.6%(229/250)	0.063

* The *p* value was compared between the two groups of 25 before and after.

**FIGURE 3:**
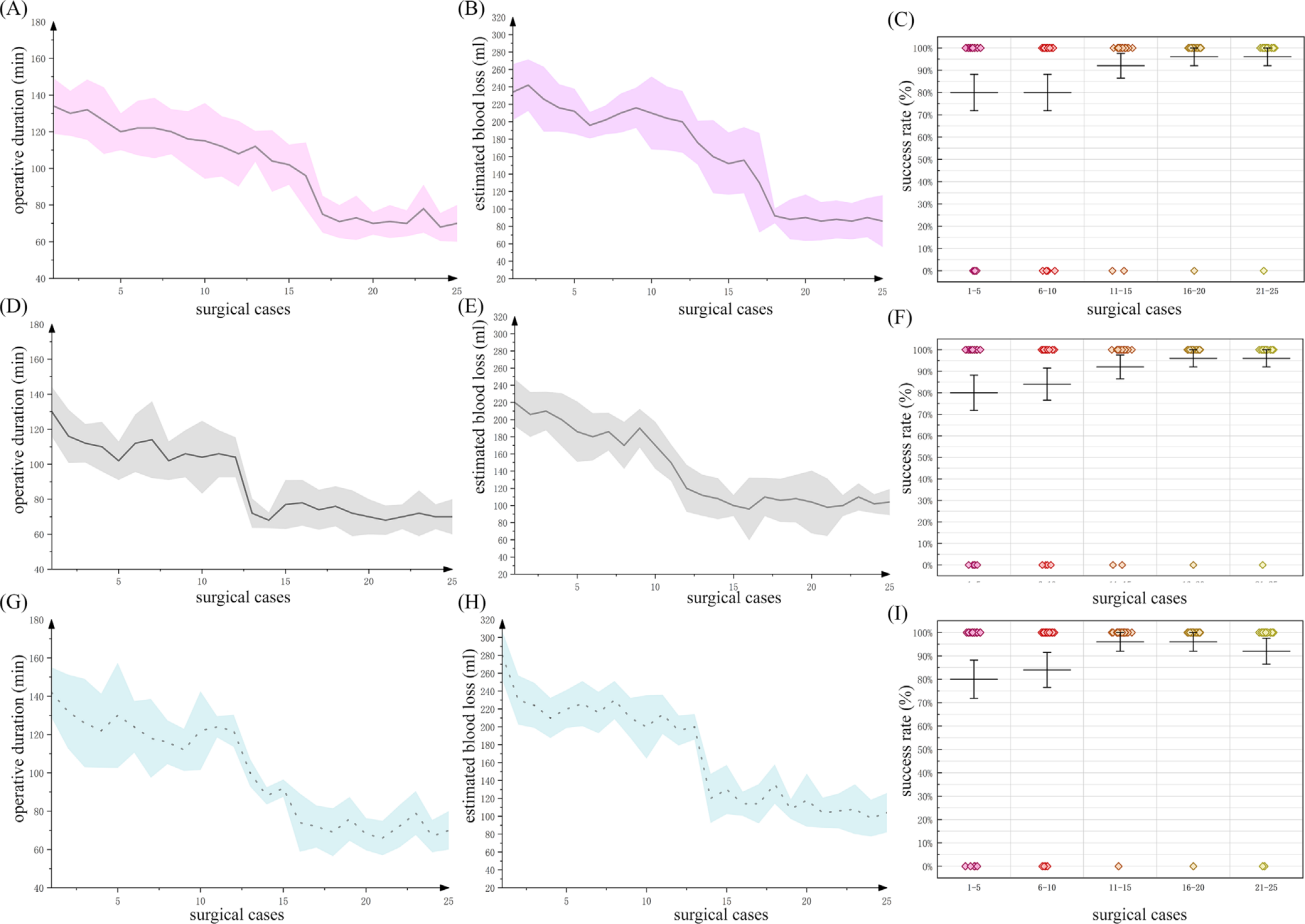
The learning curve of EPA bulbar urethroplasty in training groups. The curve shows the mean and standard deviation. Scatter plots, means, and error bars per 10 cases were used to show the surgical success rate. (A–C) Surgical time, bleeding volume, and surgical success rate in expert group as control; (D–F) surgical time, bleeding volume, and surgical success rate in fellow group; (G–I) surgical time, bleeding volume, and surgical success rate in resident group.

Before clinical practice evaluation, all the novice residents and fellows inexperienced with urethral reconstruction conducted simulator training five times in accordance with the experimental design process. In the clinical practice assessment, all patients accepted the routine operation successfully, without severe complications, such as rectum injury or infection. Finally, all the novice residents and fellows inexperienced with urethral reconstruction completed a total of 250 bulbar urethral EPA surgery (25 cases per one surgeon) independently under supervision, with an overall success rate of 90% after a 5‐year cycle (Table 2). Those groups had statistically significant differences in the estimated blood loss and operative duration. (*p* < 0.05). The uroflowmetry examination showed a similar result 6 months after the operation. In the analysis of the learning curve, the GRS scores, operative duration, estimated blood loss, and success rates in both the fellow and resident groups showed a trend toward matching or even surpassing those of the expert group (Figures 3 and S2). Compared with experts, fellow group could achieve better performance in terms of surgical time; meanwhile, resident group could achieve the success rate of surgery in a shorter period of time.

## 4 DISCUSSION

Based on the previous investigation, the average number of cases in 42% of urologic surgeons who performed urethroplasty was less than five per year. Furthermore, only 20%–29% of urologists would refer refractory strictures to another reconstructive urologist, and instead, 31%–33% would continue to manage a recurrent stricture by minimally invasive means.^9^ The learning curve of urethroplasty is believed to be influenced by the surgeon's attitude, self‐confidence, and experience. Moreover, the learning curve may be further hindered by a lack of repetition and the prolonged time interval between cases.^10^ Less practice means longer operation duration. Lacy et al.^11^ also noted that longer operation times were associated with increased rates of complications. To shorten the learning curve in the trainee, the ideal way is to have the assistance of an experienced surgeon available during early case practice.^12^ Nevertheless, the distribution of reconstructive urologists is unequal in developing and developed countries.^13^ Teaching local providers about the principles of reconstructive urology has a more sustainable impact on patient outcomes than simply conducting a high‐volume surgical trip. Above all, an ideal training simulator for urethral reconstruction may be one of the potential methods to address these key issues effectively.

In order to achieve the above ideas, we first designed and developed a simulator. When designing a new simulator, the following factors should be considered: (1) The simulator should be as realistic as possible to simulate the anatomy and pathology concerning the procedure; (2) skills learned on this model may be transferable to the operating theatre;(3) the final result of the performance can be made available for observation and feedback; (4) it may be suitable for various new practitioners, including interns, novice residents, and fellows inexperienced with urethral reconstruction; and (5) it may also be cost‐effective to produce and simple enough to be massively reproduced for a group of participants and routine use for practice. Until now, many procedure‐specific models have been reported for endourology, most of which are VR and bench models. Training modalities for laparoscopic and robot‐assisted urological surgery are mainly geared towards generic skills acquisition with a selected few procedure‐specific dry‐lab and ex vivo animal models and VR and AR platforms, respectively. In contrast, very few simulators have been produced and validated for open urological surgery.^14^ Considering the deep position and complex surrounding tissue in the perineal, we focused on designing the simulator about the bulbar urethral anastomosis instead of anterior urethroplasty.

In our study, this simulator based on the conception mentioned above was developed, and the result of face validity sufficiently supported this point. All experts had similar scores in overall satisfaction with this simulator for training beginners. In order to explore the role of simulators in urethral surgery, various new practitioners were included for observational studies. Actual equipment and instruments were utilized during the exercise. Subsequently, the final result of the anastomosis can be easily checked. Due to those points, trainees readily become familiar with and understand urethral anastomosis procedures, which increases their confidence when they perform real anastomosis in clinic practice. The results of content validation revealed those merits, as well. The results also confirm that this simulator can assess the operative proficiency of trainees afterwards. In the construction validation, we focused on determining the five critical steps of urethral anastomosis. We believed that the trainee could accomplish an accurate and simple urethral anastomosis. According to the assessment scores in our study, we have enough evidence to confirm that expert and fewer experience residents can be easily distinguished by performing this simulator. Because the anastomotic step constantly challenges an intern, we added a patency and leakage test. Through those tests, the results can verify whether the trainees can transfer from the lab to the operating room.

Simulation training offers the advantage of repetitive practice in a safe environment without compromising patient care.^15^ However, we still want to reveal whether the trainee can accomplish the actual procedure in the operation room after the training course as soon as possible. Therefore, we continued to assess the trainee's ability in the operation room. In this study, we chose the Objective Structures Assessment of Technical Skills (OSATS) global rating scale to evaluate the trainees' skills in the operation.^16^ Based on the previous reports, the OSATS scale can be applied to any assessment of surgical skills and assesses the knowledge, manipulation skill, and action record.^17^ This scale has shown to be a reliable and valid method for testing surgical skills in residents and was one of the first methods designed to evaluate competencies. Although the mean value of the OSATS scale and other parameters still showed a significant difference in each group, it seemed that the results in fellow groups tended to approach the faculty group. Our result could still reflect that the simulator can help the fellow shorten the duration of surgical proficiency fellows within a relatively short period.

There is still another limitation of this study. There is no bleeding simulated in our model. It may cause the trainee cannot deal with some complex situations, such as heavy bleeding. This issue could be improved in the operation room with more practice. In this simulator, we would instead focus on the anastomosis step and remind them of the risk of rectal injury. If those issues were trained thoroughly, most trainees could at least deal with the common bulbar urethral stricture. Secondly, the cost of the whole simulator is higher, almost $320. However, the critical part of the urethra costs only $20. The other part of the simulator can be used repeatedly. Therefore, the more trainees use the simulator, the lower price the simulator will cost. Thirdly, the design of this study mainly applied retrospective and observational studies, which may lack some credibility. In retrospective study, including those where experts were trained a decade or two ago, there are differences in the availability and sophistication of imaging, videos, and other surgical equipment. This disparity could introduce bias when plotting the learning curve. Further large‐scale prospective randomized controlled trials are needed to improve persuasiveness.

In conclusion, the synthetic training simulator for urethral anastomosis is novel, effective, convenient, and economical. It is precious for beginners in urethral reconstructive surgery. The training course can bridge the gap between lab use and actual surgery via this simulator. By this synthetic training simulator, the learning curve of urethral anastomosis could be shortened, especially both in the less experienced residents and fellows inexperienced with urethral reconstruction.

## AUTHOR CONTRIBUTIONS

*Conceptualization*: Jing‐Dong Xue, Chao Feng, and Deng‐Long Wu. *Data curation*: Hui‐Quan Shu, Lin Wang, and Wei Zhang. *Formal analysis*: Jing‐Dong Xue and Hui‐Quan Shu. *Funding acquisition*: Chao Feng and Deng‐Long Wu. *Investigation*: Ping Zhang, Ying‐Long Sa, Hong Xie, and Wei Zhang. *Methodology*: Jing‐Dong Xue, Ping Zhang, Chao Feng, and Yue‐Min Xu. *Project administration*: Ying‐Long Sa and Deng‐Long Wu. *Resources*: Ping Zhang, Yue‐Min Xu, and Chao Li. *Software*: Hui‐Quan Shu and Lin Wang. *Supervision*: Yue‐Min Xu, Ying‐Long Sa, and Deng‐Long Wu. *Validation*: Yue‐Min Xu, Hong Xie, and Chao Li. *Writing—original draft*: Jing‐Dong Xue, Ping Zhang, and Chao Feng. *Writing—review & editing*: Deng‐Long Wu, Yue‐Min Xu, and Chao Feng. All authors provided critical feedback and helped shape the research, analysis, and manuscript. All authors read and approved the final manuscript.

## CONFLICT OF INTEREST STATEMENT

The authors have no conflicts of interest to disclose.

## Supporting information


**Figure S1** The learning curve of EPA bulbar urethroplasty. Retrospective data collected from experts for the original learning curves in the early stages of their career. The case number to reach proficiency was approximately 18–20 cases for this type of reconstruction for bulbar urethroplasties, regardless of surgical time, bleeding volume, and surgical success rate. The curve shows the mean and standard deviation. Scatter plots, means, and error bars per 10 cases were used to show the surgical success rate.

**Figure S2** The Global Rating Scale of operative performance (GRS) in training groups. The curve shows the mean and standard deviation.

**Table S1** Participants' demographics and retrospective surgical experience data.

**Table S2** Results of Face and Content validity of the urethral anastomosis simulator.

**Appendix S1**. 4‐minute instructional video of training on urethral simulator.

## References

[1] Choi J , Lee CU , Sung HH . The learning curve of various types of male urethroplasty. Investig Clin Urol. 2020;61(5):508–513. 10.4111/icu.20200076 PMC7458868 32734726

[2] Faris SF , Myers JB , Voelzke BB , Elliott SP , Breyer BN , Vanni AJ , et al. Assessment of the male urethral reconstruction learning curve. Urology. 2016;89:137–142. 10.1016/j.urology.2015.11.038 26723182 PMC4792781

[3] Campain NJ , MacDonagh RP , Mteta KA , McGrath JS . Global surgery—how much of the burden is urological? BJU Int. 2015;116(3):314–316. 10.1111/bju.13170 25943037

[4] Jones CM , Campbell CA , Magee WP , Ayala R , Mackay DR . The expanding role of education and research in international healthcare. Ann Plast Surg. 2016;76(Suppl 3):S150–S154. 10.1097/SAP.0000000000000721 26808747

[5] Timberlake MD , Garbens A , Schlomer BJ , Kavoussi NL , Kern AJM , Peters CA , et al. Design and validation of a low‐cost, high‐fidelity model for robotic pyeloplasty simulation training. J Pediatr Urol. 2020;16(3):332–339. 10.1016/j.jpurol.2020.02.003 32173325

[6] Osborne MP . William Stewart Halsted: his life and contributions to surgery. Lancet Oncol. 2007;8(3):256–265. 10.1016/S1470-2045(07)70076-1 17329196

[7] Gao W , Ou T , Jia J , Fan J , Xu J , Li J , et al. Development and evaluation of a training model for paracentetic suprapubic cystostomy and catheterization. Clinics (Sao Paulo). 2019;74:e435. 10.6061/clinics/2019/e435 30994702 PMC6456918

[8] McDougall EM . Validation of surgical simulators. J Endourol. 2007;21(3):244–247. 10.1089/end.2007.9985 17444766

[9] Bullock TL , Brandes SB . Adult anterior urethral strictures: a national practice patterns survey of board certified urologists in the United States. J Urol. 2007;177(2):685–690. 10.1016/j.juro.2006.09.052 17222657

[10] Abboudi H , Khan MS , Guru KA , Froghi S , de Win G , van Poppel H , et al. Learning curves for urological procedures: a systematic review. BJU Int. 2014;114(4):617–629. 10.1111/bju.12315 24053179

[11] Lacy JM , Madden‐Fuentes RJ , Dugan A , Peterson AC , Gupta S . Short‐term complication rates following anterior urethroplasty: an analysis of national surgical quality improvement program data. Urology. 2018;111:197–202. 10.1016/j.urology.2017.08.006 28823639

[12] Zorn KC , Orvieto MA , Gong EM , Mikhail AA , Gofrit ON , Zagaja GP , et al. Robotic radical prostatectomy learning curve of a fellowship‐trained laparoscopic surgeon. J Endourol. 2007;21(4):441–447. 10.1089/end.2006.0239 17451340

[13] Burks FN , Salmon SA , Smith AC , Santucci RA . Urethroplasty: a geographic disparity in care. J Urol. 2012;187(6):2124–2127. 10.1016/j.juro.2012.01.078 22503011

[14] Aydin A , Raison N , Khan MS , Dasgupta P , Ahmed K . Simulation‐based training and assessment in urological surgery. Nat Rev Urol. 2016;13(9):503–519. 10.1038/nrurol.2016.147 27549358

[15] Lima JCS , Rocha HAL , Mesquita FJC , Araújo DABS , Silveira RA , Borges GC . Simulated training model of ureteropyelic anastomosis in laparoscopic pyeloplasty. Acta Cir Bras. 2020;35(11):e351108. 10.1590/acb351108 33331458 PMC7748080

[16] MacEwan MJ , Dudek NL , Wood TJ , Gofton WT . Continued validation of the O‐SCORE (Ottawa Surgical Competency Operating Room Evaluation): use in the simulated environment. Teach Learn Med. 2016;28(1):72–79. 10.1080/10401334.2015.1107483 26787087

[17] Pinheiro EFM , Barreira MA , Moura Junior LG , Mesquita CJG , Silveira RA . Simulated training of a laparoscopic vesicourethral anastomosis. Acta Cir Bras. 2018;33(8):713–722. 10.1590/s0102-865020180080000007 30208133

