# Supplementary figures and images for: Clinical application values of a novel synthetic training simulator for bulbar urethral anastomosis
Source: BJUI Compass. 2024 Aug 30;5(10):916–23. doi: 10.1002/bco2.426 (PMC11479804; doi:10.1002/bco2.426)

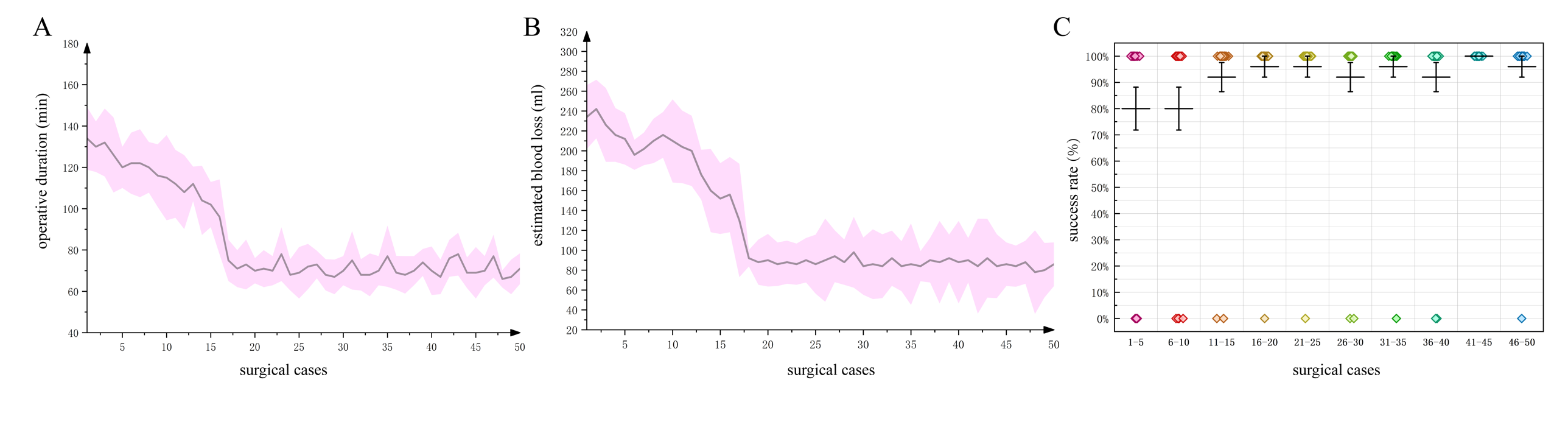

Supplement: Supplementary file 1 — Figure S1 The learning curve of EPA bulbar urethroplasty. Retrospective data collected from experts for the original learning curves in the early stages of their career. The case number to reach proficiency was approximately 18–20 cases for this type of reconstruction for bulbar urethroplasties, regardless of surgical time, bleeding volume, and surgical success rate. The curve shows the mean and standard deviation. Scatter plots, means, and error bars per 10 cases were used to show the surgical success rate. [file BCO2-5-916-s005.tif]

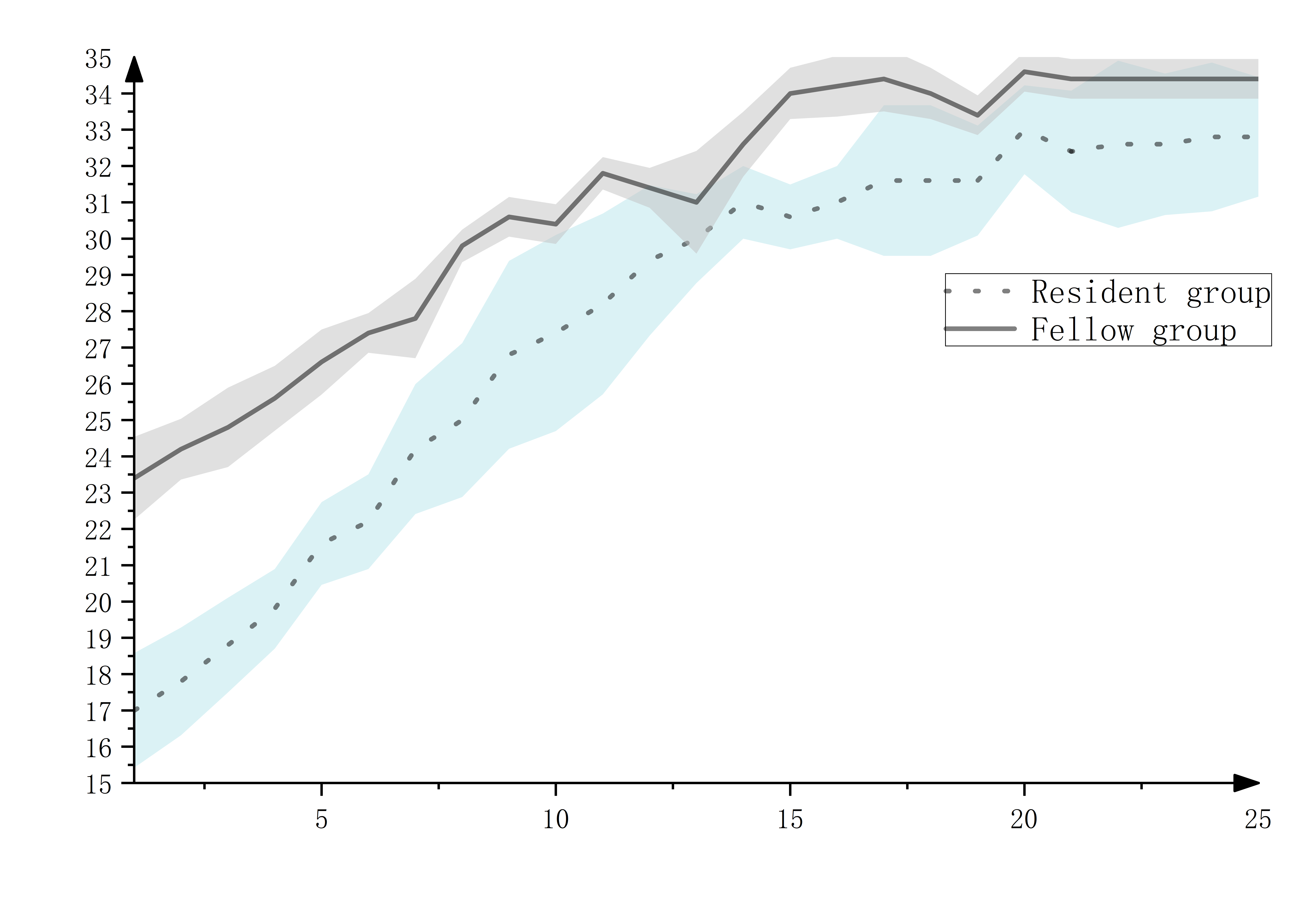

Supplement: Supplementary file 2 — Figure S2 The Global Rating Scale of operative performance (GRS) in training groups. The curve shows the mean and standard deviation. [file BCO2-5-916-s004.tif]
