# Supplementary material for: Clinical application values of a novel synthetic training simulator for bulbar urethral anastomosis
Source: BJUI Compass. 2024 Aug 30;5(10):916–23. doi: 10.1002/bco2.426 (PMC11479804; doi:10.1002/bco2.426)
Supplement: Supplementary file 3 — Table S1 Participants' demographics and retrospective surgical experience data. [file BCO2-5-916-s002.docx]

| Table S1 Participants’ demographics and retrospective surgical experience data | | | |
| --- | --- | --- | --- |
|  | Fellow (n=5) | Resident (n=5) | Intern (n=10) |
| Age, yrs | 37 (33-40) | 31 (29-33) | 25 (24-28) |
| Sex(male/female) | 5/0 | 5/0 | 9/1 |
| Surgical experience beyond training, yrs | 8 (5-13) | 2 (0-3) | 0 (0-0) |
| Urethroplasty assistant experience beyond training, yrs | 5 (0-20) | 5 (0-15) | 2 (0-4) |
|  | Expert (n=5) |  |  |
| Estimated total number of urethroplasty performed | 800 (500-2000) | / |  |
| Estimated total number of EPA performed | 700 (200-1000) | / |  |
| Number of urethroplasty performed annually | 100 (70-200) | / |  |
| Number of EPA performed annually | 50 (30-80) | / |  |
| All statistics are reported as median (range) |  |  |  |
|  |  |  |  |
