# Supplementary material for: Clinical application values of a novel synthetic training simulator for bulbar urethral anastomosis
Source: BJUI Compass. 2024 Aug 30;5(10):916–23. doi: 10.1002/bco2.426 (PMC11479804; doi:10.1002/bco2.426)
Supplement: Supplementary file 4 — Table S2 Results of Face and Content validity of the urethral anastomosis simulator. [file BCO2-5-916-s001.docx]

| Table S2. Results of Face and Content validity of the urethral anastomosis simulator | | |
| --- | --- | --- |
| Face and Content Validity of Realism and Usefulness of the simulator  (1=not very realistic or not suitble at all to 5=Very Realistic or suitble) | | |
|  | Expert A | Expert B |
| Anatomy and color of the model | 4 | 4 |
| Sensation of texture and feeling of cuting | 4 | 4 |
| Conductibility of suturing | 5 | 5 |
| Overall satisfaction of the model | 5 | 5 |
| Is this a useful model for teaching urethral anastomosis | 5 | 5 |
| Did you gain transferable skills to operating theater from this model | 4 | 4 |
| Do you feel more confident in performing urethral anastomosis after having practice on this model | 5 | 4 |
| Do you think this will help to improve trainee’s confidence in performing urethral anastomosis | 4 | 5 |
| Do you think this model can be used as routine training for urethral anastomosis | 5 | 5 |
